# Acute pain impairs retention of locomotor learning

**DOI:** 10.1152/jn.00343.2023

**Published:** 2024-02-21

**Authors:** Jessica E. Galgiani, Margaret A. French, Susanne M. Morton

**Affiliations:** ^1^Department of Physical Therapy, University of Delaware, Newark, Delaware, United States; ^2^Interdisciplinary Graduate Program in Biomechanics and Movement Science, University of Delaware, Newark, Delaware, United States

**Keywords:** capsaicin, consolidation, locomotion, motor learning, walking

## Abstract

Despite abundant evidence that pain alters movement performance, considerably less is known about the potential effects of pain on motor learning. Some of the brain regions involved in pain processing are also responsible for specific aspects of motor learning, indicating that the two functions have the potential to interact, yet it is unclear if they do. In *experiment 1*, we compared the acquisition and retention of a novel locomotor pattern in young, healthy individuals randomized to either experience pain via capsaicin and heat applied to the lower leg during learning or no stimulus. On *day 1*, participants learned a new asymmetric walking pattern using distorted visual feedback, a paradigm known to involve mostly explicit re-aiming processes. Retention was tested 24 h later. Although there were no differences in *day 1* acquisition between groups, individuals who experienced pain on *day 1* demonstrated reduced retention on *day 2*. Furthermore, the degree of forgetting between days correlated with pain ratings during learning. In *experiment 2*, we examined the effects of a heat stimulus alone, which served as a control for (nonpainful) cutaneous stimulation, and found no effects on either acquisition or retention of learning. Thus, pain experienced during explicit, strategic locomotor learning interferes with motor memory consolidation processes and does so most likely through a pain mechanism and not an effect of distraction. These findings have important implications for understanding basic motor learning processes and for clinical rehabilitation, in which painful conditions are often treated through motor learning-based interventions.

**NEW & NOTEWORTHY** Pain is a highly prevalent and burdensome experience that rehabilitation practitioners often treat using motor learning-based interventions. Here, we showed that experimental acute pain, but not a heat stimulus, during locomotor learning impaired 24-h retention of the newly learned walking pattern. The degree of retention loss was related to the perceived pain level during learning. These findings suggest important links between pain and motor learning that have significant implications for clinical rehabilitation.

## Graphical abstract

**Figure:**
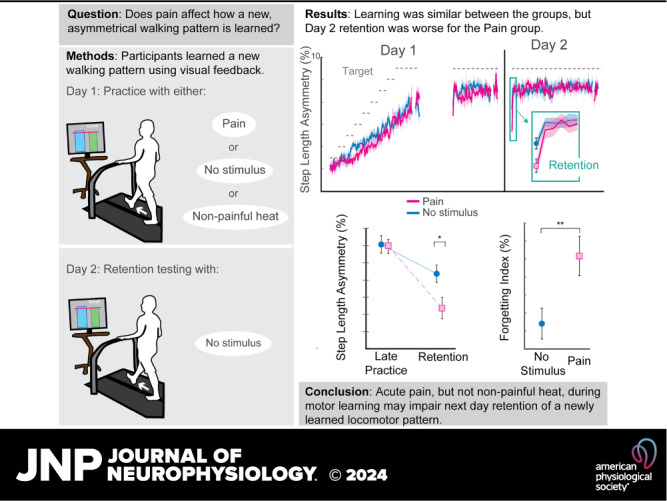

## INTRODUCTION

Pain is highly prevalent and extremely burdensome for those who experience it (1). It is estimated that half of adults in the United States will experience at least some pain in any given 3-mo period, with over 17% of adults reporting that they experience pain most or every day (2). To minimize pain and combat disability, physical rehabilitation is commonly prescribed (3). Much of physical rehabilitation is based on motor learning principles (4) in which better or more appropriate movement patterns are learned using task-specific practice (5); e.g., teaching an assembly line worker how to lift a load with improved body mechanics to help resolve back pain and lower the risk of injury. Considering that rehabilitation is one of the few nonpharmacologic treatment options for people living with pain, understanding how and to what degree acute or chronic pain affects motor learning is crucial to the realization of successful rehabilitation outcomes for these individuals. Although it is clear that acute pain can change muscle activation patterns and specific movement strategies, i.e., motor performance (6–11), much less is known about how pain may impact the ability to acquire and retain new motor patterns; i.e., motor learning, and retention of motor learning.

Numerous fMRI and PET studies have shown that acute and chronic pain produce significant activation changes in multiple brain regions. This “pain network” extends well beyond somatosensory areas into key motor regions, including the primary motor (M1), premotor, supplementary motor, anterior cingulate, and prefrontal cortices (12–14). In addition, chronic pain results in structural changes to many of these same regions (15–16). More direct support for a relationship between pain and M1 activation comes from brain stimulation studies. Increasing M1 excitability reduces pain (17, 18), and the presence of an acute painful stimulus decreases M1 excitability (19–21). From the motor learning literature, it has been shown that increasing or decreasing M1 excitability results in increased (22) or decreased (23–25), respectively, motor memory consolidation of newly learned movements. Here, consolidation refers to the neural processes whereby new motor memories are made less labile, enabling their storage for long-term use and retention (26, 27). Putting these together, pain is associated with decreased M1 excitability, and decreased M1 excitability is associated with reduced motor memory consolidation. In fact, maladaptive activity in any number of brain regions affected by acute or chronic pain, including M1, prefrontal cortex, premotor cortex, and/or parietal cortex, could affect motor consolidation (22, 24, 25, 28).

In humans, a small body of literature supports a relationship between pain and motor learning specifically. For example, noxious capsaicin cream applied to the tongue during the acquisition of a novel intraoral tongue-pressing task reduces practice-based performance gains, an apparent learning deficit (29). Also, intramuscular hypertonic saline in the deltoid muscle decreases the rate of reaching performance gains (30), and tonic cutaneous pain applied to the lower leg reduces 24-h retention of a locomotor adaptation (31). However, these findings have not been consistently replicated (32–35) and limitations in study design have been noted. Specifically, in the majority of the prior work, motor performance changes have not been distinguished from motor learning changes during painful conditions. That is, if performance of the task to be learned is sufficiently painful or limited, what may appear to be an inability to learn may instead actually reflect an inability or unwillingness to perform a new movement. For example, in the tongue-pressing task, participants experiencing pain used less force and spent less time in the target zone, probably because pressing the key increased their tongue pain (29), and in the arm reaching study, participants with pain changed their muscle recruitment strategy (30). In these studies, and others, it is unclear if pain truly interfered with learning, which we define as the capacity to acquire a novel movement pattern through task practice, or simply resulted in people choosing to move differently to avoid or reduce the pain.

Furthermore, it is critical to examine the effect of pain on retention over days. Not only is retention more relevant to rehabilitation, but there is also the aforementioned physiological evidence supporting a relationship between pain and reduced activation in brain regions supporting motor memory consolidation following motor learning (19–21). Therefore, the goal of the present study was to investigate the effect of acute pain on the acquisition and retention of a new walking pattern. We used a locomotor learning paradigm developed in our laboratory that uses real-time visual feedback to drive motor learning of a novel step asymmetry (36, 37) and engages a primarily explicit, strategic form of motor learning, which is consistent with how new movement patterns are taught in clinical rehabilitation settings. To reduce the potential confound of pain interfering with motor performance, we used a tonic, cutaneous pain stimulus that remains constant regardless of movement. In *experiment 1*, we compared the acquisition and retention of a novel step length asymmetry pattern during walking between individuals who received a painful stimulus during the initial learning and those who had no pain. We suspected that the effects of acute pain would specifically disrupt the processes associated with motor memory consolidation, thus we hypothesized that acute pain would result in impaired retention of this new gait pattern 24 h later but not significantly affect the initial acquisition of a novel walking asymmetry (H1). We also hypothesized that the degree of pain perceived would be positively correlated with this retention impairment (H2). Based on the results of *experiment 1* and to examine whether differences between pain and no pain groups could be due to an effect of attention or distraction, we conducted *experiment 2* to test the effects of a tonic, nonpainful heat stimulus applied during learning. We hypothesized that there would be no effect of nonpainful heat on locomotor learning acquisition or retention (H3).

## METHODS

### Participants and Groups

Young, healthy pain-free participants were recruited via flyers posted in and around a university campus and in online classified ads. Enrollment criteria were: age 18–35 yr, no acute or chronic pain, not taking any analgesics, no musculoskeletal or neurological conditions, naïve to the experimental design, and willingness to receive the experimentally induced pain. Participants were also excluded if they had any condition that could result in an adverse reaction to experimental pain, including decreased sensation or circulation in the area targeted for the pain stimulus, any skin lesion in the area, or skin allergies. For *experiment 1*, 25 individuals were recruited and randomized into one of two groups, Pain (*n* = 12) or No Stimulus (*n* = 13). Those in the Pain group received a temporary, harmless, painful stimulus experimentally induced with capsaicin and heat during the learning portion on *day 1* only, whereas those in the No Stimulus group received nothing. Based on our findings from this experiment, we then conducted *experiment 2*, in which a Heat Only (*n* = 12) group was recruited and enrolled to experience nonpainful heat during the learning on *day 1*, to serve as a tonic somatosensory and attentional stimulus control. All participants provided written informed consent and this protocol was approved by the University of Delaware IRB.

### Paradigm

**Figure 1.**
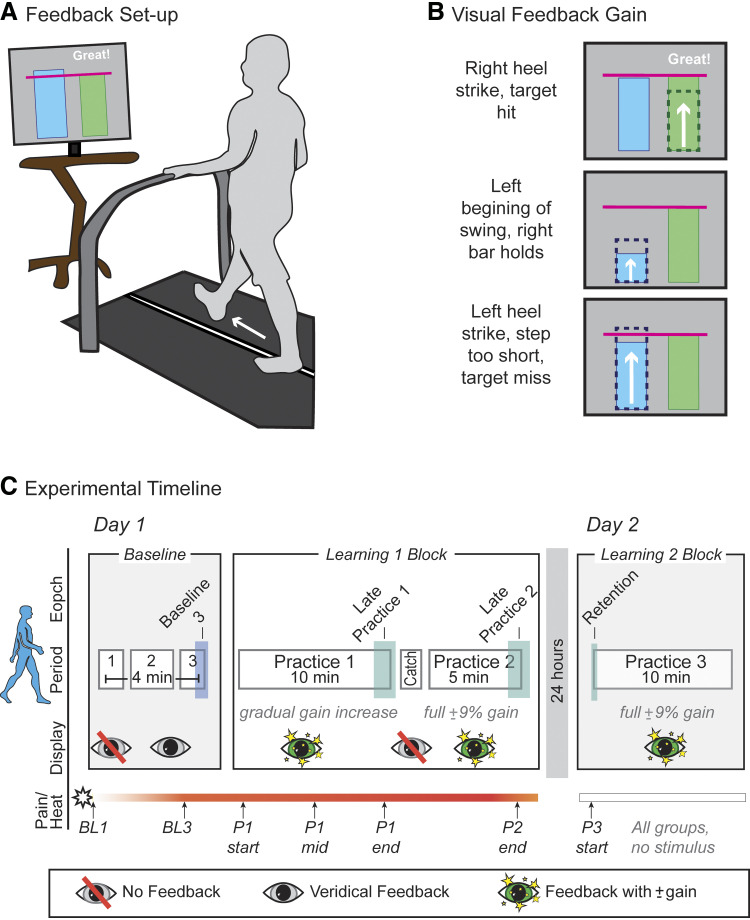
Experimental paradigm. *A*: treadmill walking setup: participants walked on a treadmill while viewing visual feedback of their step lengths on a monitor. Step lengths were depicted as vertical bars that grew in height in real time with each step. *B*: visual feedback: during *practice* periods, feedback was altered to drive learning of a novel asymmetric stepping pattern. Dotted line bars indicate the lengths of step actually taken (not visible to participants); solid bars indicate the step lengths with the gain included (shown to participants). Here, three sample time periods are shown for illustration purposes, but the visual feedback was provided continuously in real time. *C*: experimental timeline: blue and green shading indicate the key epochs during *baseline*, *learning 1*, and *learning 2* blocks used for analysis. Participants were asked to rate their pain at the time points indicated with vertical arrows (pain ramp-up ratings not shown). BL, baseline; P, practice.

During *day 1*, participants acquired (learned) a new asymmetric stepping pattern while walking on a treadmill. On *day 2*, participants were tested on their retention of the newly learned stepping pattern. During both days, treadmill belt speeds were set to 1.1 m/s for all participants. A ceiling-mounted harness (providing no body weight support) was donned, and participants were instructed to lightly hold onto a front handrail during walking for safety. During some portions of walking, real-time visual feedback was displayed (The Motion Monitor Toolbox, Innovative Sports Training Inc., Chicago, IL) on a computer monitor (50 cm) located at eye level approximately 125 cm in front of the participant (see Fig. 1*A*). Participants learned a novel asymmetric step length pattern by responding to a gain added to the visual feedback provided by the real-time display.

#### Visual display.

All participants viewed the same feedback display, which consisted of a dynamic graph with two vertical bars, side by side, one for each leg. The height of each bar represented the instantaneous step length of the corresponding leg throughout the swing phase, scaled to the size of the screen. That is, at the initiation of swing, the bar would grow vertically from the bottom of the screen (height = 0) until the time of the next heel strike of that foot (height = full step length). It would then hold its position at that height until the next step with that leg began. This continued for both legs so that participants received feedback on the length of each step on each side as they walked. A horizontal line was generated from each participant’s average baseline step length and represented the target step length to be achieved. When this line was visible on the screen, participants were instructed to “hit the target” by making their steps match the target step length. For each correct target hit, a word would appear on the screen, e.g., “Great!,” to alert participants that they had been successful. The target line was set with a margin of error of ±1.5% of the baseline average. During the practice periods (see *Experimental timeline* section), the visual feedback was altered to drive learning of a novel step length asymmetry. Specifically, a gain was added to the height of the bars. The left bar showed participants taking 9% shorter steps than their true left step length (−9% gain) and the right bar showed participants taking 9% longer steps than their true right step length (+9% gain) while the horizontal target line remained at the baseline step length (Fig. 1*B*). Therefore, to hit the targets, participants had to adopt a 9% walking asymmetry. Despite the alteration of the feedback, recent work in our laboratory suggests that learning this walking task relies predominantly on explicit, strategic re-aiming processes and not implicit sensorimotor adaptation (36, 37).

#### Experimental timeline.

The timeline, which was identical for both *experiments 1* and *2*, is illustrated in Fig. 1*C*. *Day 1* consisted of *baseline* (3–4 min) and *learning 1* (15 min) blocks with each block made up of several periods. Between each period, the treadmill was stopped for 30–60 s so instructions for the next period could be provided. The *baseline* block was subdivided into *baseline 1*, *2,* and *3* periods. In *baseline 1* (1 min), participants walked without visual feedback to acclimate to the treadmill and to obtain baseline step length measurements. In *baseline 2* (1–2 min), participants were oriented to the visual display by viewing veridical step length feedback. They were encouraged to try taking steps of different lengths to understand how the display reflected their own real-time step lengths. All participants were required to demonstrate understanding of the display by correctly taking a longer/shorter step when prompted before they could proceed to the next baseline period. In *baseline 3* (1 min), participants were asked to “look straight ahead and walk normally” while viewing veridical visual feedback on the display. The *learning 1* block was subdivided into *practice 1*, *catch,* and *practice 2* periods. Prior to the beginning of *practice 1*, all participants were told to “try and hit the target” with both legs while walking, but were not informed of the upcoming change in step length symmetry required or specifically what they would need to do to be successful at the task. During *practice 1* (10 min), participants learned the new asymmetric stepping pattern using the visual feedback with the ±9% gain as described earlier. Throughout this period, participants were asked to hit the target with each step by making the bars reach but not exceed the horizontal line representing the average step length during *baseline 1*. The gain in the feedback for each leg was introduced gradually over 8 min, growing from a step length asymmetry of 1% for the first minute and increasing by 1% each minute. We chose to gradually introduce the gain change, as opposed to introducing it all at once at the beginning of *practice 1*, so that participants would be forced to continuously learn and adjust their step symmetry throughout the entire period and not be able to learn the full pattern in just the first few minutes and then simply repeat that pattern. During the final 2 min of *practice 1*, the visual feedback from the bars was held at 9% asymmetry. Between *practice 1* and *practice 2* periods was the *catch* period (20 s). During this time, all visual feedback on the display was removed and participants were instructed to “look straight ahead and walk normally.” The purpose of the *catch* period was to assess, after 10 min of learning, how much of the learning had been implicitly stored (36–38). In *practice 2* (5 min), the full ±9% gain was returned to the visual feedback. The purpose of *practice 2* was to restore any learning that may have been forgotten or “unlearned” during the brief *catch* period. Participants were instructed to “hit the target with each step, just as you did before.” The end of the *practice 2* period marked the end of *day 1* testing (see Fig. 1*C*). Participants were instructed to avoid use of a treadmill between testing days.

Approximately 24 h after *day 1*, participants returned to the laboratory for *day 2* retention testing. Here, a single 10-min *learning 2* block (*practice 3* period) was performed, in which participants walked with the same ±9% gain in the visual feedback that they experienced during the end of the *practice 1* period and all of the *practice 2* period on *day 1*. As during *day 1* practice, they were instructed to “hit the target” with each step.

#### Pain and heat stimuli.

For *experiment 1*, participants in the Pain group received a harmless but painful stimulus induced by combining capsaicin cream with heat on the skin. During *day 1*, the skin was cleaned with alcohol and a 0.1% topical preparation of capsaicin was applied generously to an 8-cm band of skin around the right mid-shank, approximately halfway between the level of the malleoli at the ankle and inferior pole of the patella. Capsaicin at this low concentration is not typically painful, but it does make the skin more sensitive to heat (39–41). Therefore, to induce pain, a disposable, air-activated heat wrap was applied over the capsaicin-treated skin and secured with hypoallergenic tape. We selected capsaicin and heat as our experimental pain model, as opposed to a musculoskeletal form of pain such as intramuscular hypertonic saline injection, because tonic cutaneous pain is not affected by muscle contraction or limb movement and is therefore unlikely to result in motor performance-related changes. In this way, we avoided the possible confound of motor performance changes (alteration or unwillingness to change a movement pattern due to pain) appearing as learning deficits. Participants completed *baseline 1* and then sat and rested quietly while the temperature of the heat wrap gradually increased to its tonic level (“ramp up” period). The pain stimulus was considered to be at full strength when *1*) the participant-rated pain level did not increase for three consecutive measurements or *2*) pain was rated at a 7/10 (see *Pain measures* section for pain rating assessment details). Time to reach full strength ranged from 10 to 35 min, and once achieved, the *baseline 2* period began. The capsaicin with the heat wrap was worn for the remainder of *day 1*. Immediately following the *learning 1* block on *day 1*, the heat wrap was removed, and the treated skin was gently cleansed with mild soap and cool water. Participants in the No Stimulus group had nothing applied to their leg, but completed a seated rest break, similar to the Pain group, for 5–10 min.

For *experiment 2*, participants in the Heat Only group experienced the same heat stimulus from the heat wrap as the Pain group, but without the capsaicin cream. The heat wrap was worn during the same periods as the Pain group. This group served as a test for any potential effects caused by non-noxious somatosensory inputs, e.g., any effect of attention or distraction caused by a warm sensation on one leg. Note that the temperature of the heat wrap is just below the threshold for heat pain for this size and location of stimulus (∼40–45°C), so it is a relatively strong stimulus (42), as strong as could be safely delivered without inducing pain. All participants in all groups were pain-free prior to leaving the laboratory on *day 1* and were pain-free throughout *day 2*.

### Data Collection

#### Motion capture measures.

Participants walked on a custom-built, in-ground, dual-belt treadmill (Bertec, Columbus, OH) with imbedded force plates under each belt. Kinematic data were collected with an 8-camera three-dimensional Vicon MX40 motion capture system (Vicon Motion Systems, Inc., Centennial, CO). Reflective markers were placed on the heel, lateral aspect of the 5th metatarsal head, and the lateral malleolus, bilaterally, as well as an additional marker on the left medial metatarsal head only. This asymmetrical configuration enhanced the real-time fidelity of the visual feedback provided to participants. The real-time visual feedback display used these position data to create the step length bar graphs. Kinematic data were recorded at a frequency of 100 Hz. Ground reaction forces were recorded from the force plates at a frequency of 1,000 Hz and time-synchronized with the kinematic data in Vicon Nexus software.

#### Pain measures.

Regardless of group assignment, all participants were regularly asked to rate their pain using a numeric scale spanning whole numbers from 0 (no pain) up to 10 (maximum possible pain imaginable). This is considered a valid and reliable method for assessing subjective pain levels (43). We used a graphic with verbal anchors and descriptors as well as colors and cartoon facial expressions to teach participants how to use this scale and select the whole number that most closely matched their pain experience. Pain levels were assessed every 3–5 min during pain ramp-up (or heat only or rest) period and every 5 min during the *baseline 3* period and the *learning 1* block on *day 1*, and once at the beginning of testing on *day 2*.

### Data Analysis

Data were analyzed with custom-written MATLAB code (MathWorks, Natick, MA). Kinematic and kinetic data were each low-pass filtered at 10 Hz. Gait events were detected with a combination of marker position and force-plate data. Initial contact (heel strike) was determined as the first time point in which the vertical ground reaction force exceeded 20 N on the corresponding treadmill belt after the anterior-posterior velocity of the heel marker first changed direction. Right and left step lengths were defined as the anterior-posterior distance between the two heel markers at the time of right and left initial contact, respectively.

Our chief outcome measure was step length asymmetry (SLA), which has been used previously to quantify learning in this paradigm (36, 37). For each stride, SLA was defined as

$$\text{SLA}=\left(\frac{{\text{Step Length}}_{\text{Left}}-{\text{Step Length}}_{\text{Right}}}{{\text{Step Length}}_{\text{Left}}+{\text{Step Length}}_{\text{Right}}}\right)\times 100\%$$

For this calculation, a value of zero represents perfect symmetry, and positive values indicate a longer step was taken with the left leg. In this paradigm, perfect learning in response to the altered visual feedback would produce a SLA value of +9%.

We focused our analysis on three key epochs: Late Practice 1 (the average SLA over the last 25 strides of the *practice 1* period), Late Practice 2 (the average SLA over the last 25 strides of the *practice 2* period), and Retention (the average SLA over the first 4 strides of the *practice 3* period on *day 2*). The Late Practice 1 and Late Practice 2 epochs were used to quantify acquisition (initial learning), and the Retention epoch was used to quantify 24-h retention. We further quantified retention by calculating a forgetting index that was the change in SLA from the end of *day 1* (Late Practice 2 epoch) to the beginning of *day 2* (Retention epoch).

#### Statistical analysis.

Statistical analyses were conducted using SPSS (IBM Corporation, Armonk, NY, v.25.0) except where noted. For all comparisons, the α level was set to 0.05. Assumptions of normality, sphericity, and equality of variance were tested with Shapiro–Wilk, Mauchly, and Levene tests, respectively. Individuals with incomplete data were excluded from analysis. For *experiment 1*, we first used equivalency testing, in the form of the two one-sided *t* test (TOST) procedure (44), to examine whether the Pain and No Stimulus groups performed similarly during the end of *baseline 3* (average of the last 25 strides), despite the Pain group experiencing pain during that time. We considered any difference between the two groups that was within 1.5% SLA to be sufficiently small to be considered equivalent, given that ±1.5% of an individual’s step length was the margin of error for step target hits during practice periods. We used the TOSTER spreadsheet to perform this analysis (44). For the main analysis testing H1, we compared learning acquisition and retention levels between Pain and No Stimulus groups using a 2 × 3 mixed design ANOVA with repeated measures on the factor “epoch” (Late Practice 1, Late Practice 2, Retention). We followed any significant interaction effects with planned pairwise comparisons [(Fisher’s least significant difference (LSD)]. Forgetting indices for each group were compared using a Student’s independent *t* test. To determine whether there were any relationships between pain and locomotor learning acquisition, retention, or forgetting (test of H2), we computed a mean pain score for each participant in the Pain group as the average of all pain ratings taken during *baseline 3* and *practice 1* and *2* periods. We used Spearman’s rank coefficients to determine the correlation between mean pain rating and SLA values at the Late Practice 2 and Retention epochs as well as forgetting index. For *experiment 2* (test of H3), we repeated the exact same 2 × 3 ANOVA with planned comparisons and the independent *t* test as described earlier for *experiment 1*, this time comparing between No Stimulus and Heat Only groups.

## RESULTS

### Participant Demographics

A total of 37 young, healthy individuals participated in this study. Data from *day 2* were not available for two participants in the No Stimulus group, due to technical difficulty. Data from one participant in the Pain group were excluded because this participant verbalized that they did not attempt to follow the examiner’s instructions during the learning task. This left 11 people in the No Stimulus group (8 females; mean age ± 1 SD, 21.5 ± 1.97 yr), 11 people in the Pain group (5 females; age 22.6 ± 1.29 yr), and 12 people in the Heat Only group (3 females; age 24.4 ± 3.29 yr).

### Experiment 1

#### Reported pain levels.

**Figure 2.**
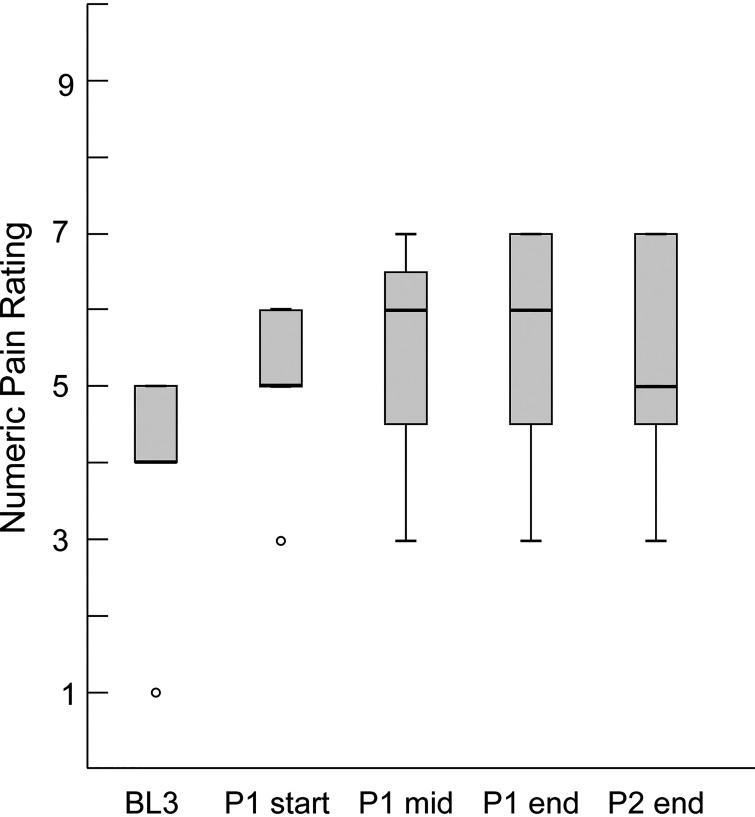
*Experiment 1*. *Day 1* pain ratings for the Pain group. Black horizontal bars indicate median pain ratings, shaded boxes indicate the interquartile ranges, and lower and upper whiskers indicate the minimum and maximum ratings, respectively. Open circles indicate outliers. BL, baseline; P, practice.

All participants in the No Stimulus group rated their pain as 0 at all time points. Figure 2 shows pain ratings for participants in the Pain group. All these individuals initially rated their pain as 0/10 prior to application of the painful stimulus. Moderate levels of pain were reported after ramp-up and maintained during the walking on *day 1*, with a median reported pain of 5/10 at the end of the *practice 2* period (Fig. 2). All participants rated their pain as 0/10 after removal of the pain stimulus, as well as on *day 2*.

#### Baseline walking performance with pain.

Average SLA at Late Baseline 3 was very small for both groups (No Stimulus group: −0.29% ± 1.5% vs. Pain group: −0.28% ± 1.0%) and the TOST procedure indicated that the two groups were equivalent [*t*(17.7) = −2.7, *P* = 0.007). Thus, baseline stepping performance was equivalent with or without the presence of the painful stimulation.

#### Locomotor learning acquisition and retention (H1).

**Figure 3.**
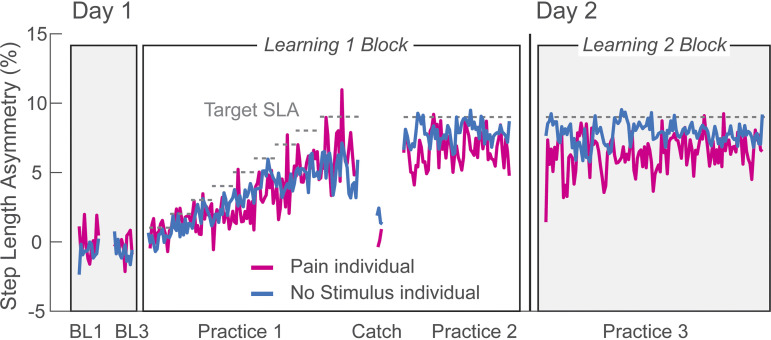
*Experiment 1*. Step length asymmetry values (SLAs) shown on a stride-by-stride basis (in bins of 4 strides) for two exemplar participants, one each from the Pain (pink trace) and No Stimulus (blue trace) groups. Gray dashed horizontal lines indicate the target SLA. BL, baseline.

**Figure 4.**
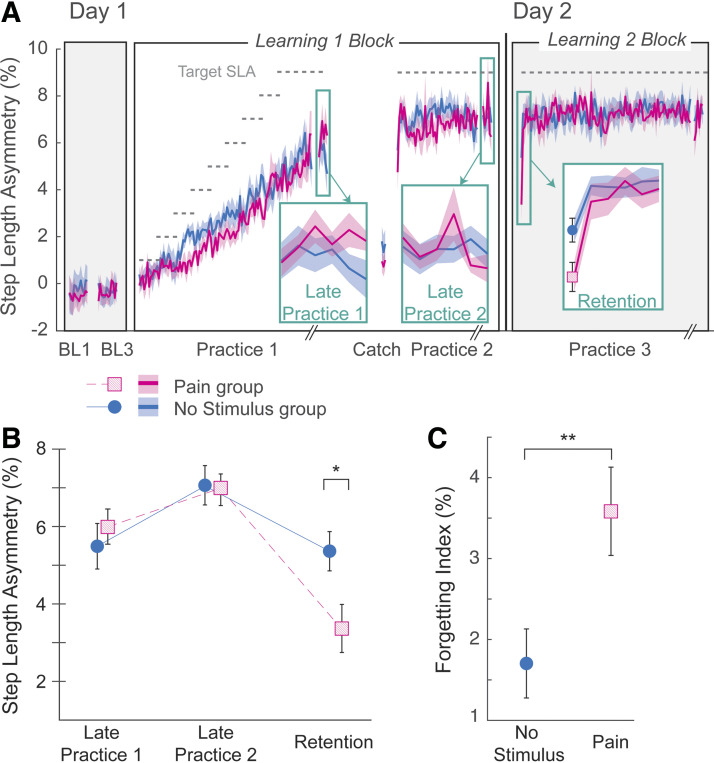
*Experiment 1*. *A*: group average step length asymmetry values (SLAs) shown on a stride-by-stride basis (in bins of 4 strides) for the Pain group (pink trace) and No Stimulus group (blue trace). Data are truncated to the length of the individual with the shortest Practice period, with the final six bins shown for all (indicated by hatching on the *x*-axis). Gray dashed horizontal lines indicate the target SLA. Insets show the Late Practice 1, Late Practice 2, and Retention epochs. Shading indicates ±1 SE. BL, baseline. Group average SLAs at Late Practice 1, Late Practice 2, and Retention epochs (*B*), and group average forgetting indices for the Pain (pink squares) and No Stimulus (blue circles) groups (*C*). Error bars indicate ±1 SEM. *Significant pairwise comparison post hoc from the interaction effect. **Significant difference from the independent *t* test between groups.

Figure 3 shows stride-by-stride plots of SLAs during *day 1* and *day 2* from representative individuals from the Pain and No Stimulus groups. During *practice 1*, the participants learned to increase their SLA following the gradual increase of the gain in the visual feedback. Both participants appeared to learn similarly during *practice 1* and *2* periods on *day 1*. On *day 2*, the participant in the Pain group started off with a smaller asymmetry during the *learning 2* block (Retention epoch) compared with the participant in the No Stimulus group, but by the end of *day 2*, both participants appeared similar again. Group average SLA values are presented on a stride-by-stride basis in Fig. 4*A*. Results are comparable with the individual data in that learning acquisition on *day 1* was similar between groups, but the initial Retention epoch differed (see insets). Group average SLAs are shown in Fig. 4*B* for the Late Practice and Retention epochs. For the mixed ANOVA comparing the two groups across the epochs Late Practice 1, Late Practice 2, and Retention, all assumptions were satisfied. There was a significant group × epoch interaction effect [*F*(2,40)= 7.8, *P* = 0.001, $\eta_{\text{p}}^{2}$ = 0.28], which we followed with planned pairwise comparisons. There were no differences between groups at either the Late Practice 1 or Late Practice 2 epochs (*P* = 0.467, *P* = 0.862, respectively) but there was a significant difference between groups at the Retention epoch (*P* = 0.022). Mean SLA at the Retention epoch for the Pain group (3.4% ± 2.1 SD) was only ∼60% of the mean SLA of the No Stimulus group (5.4% ± 1.7 SD), indicating that 24-h retention was reduced in the Pain group.

This finding was corroborated with the forgetting index values (Fig. 4*C*). Recall that the forgetting index is a unique measure from the SLA value at the Retention epoch because the forgetting index takes into account, for each participant, the learning achieved on the prior day. Here, the No Stimulus group average forgetting was about 1.7% SLA, thus forgetting about a quarter of what they had learned. The Pain group average forgetting was about 3.6% SLA, or about half of what they had learned, which was significantly more than the No Stimulus group [*t*(20) = 2.7, *P* = 0.013, *d* = 1.17].

#### Correlations between pain perception and locomotor learning (H2).

**Figure 5.**
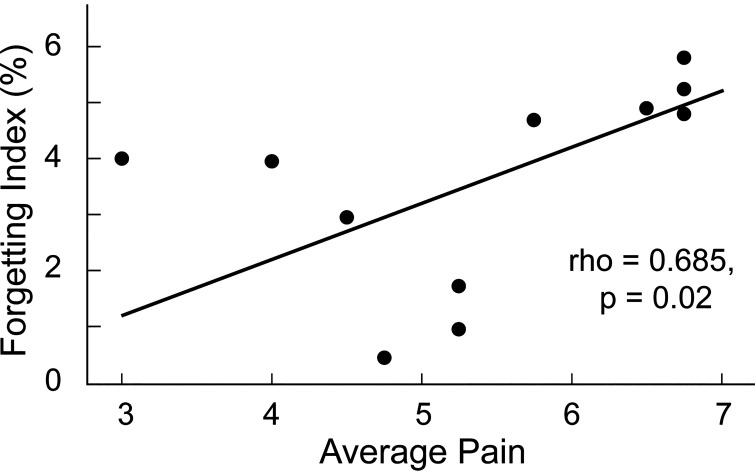
*Experiment 1*. Scatterplot showing the correlation between average pain rating and forgetting index for the Pain group.

In addition, we found a moderately strong and significant correlation between mean pain ratings and forgetting indices (Spearman’s ρ = 0.685, *P* = 0.020, Fig. 5) for participants in the Pain group. That is, participants who experienced higher levels of pain during learning tended to forget more of the walking pattern than participants who experienced lower levels of pain, with the five people who forgot the most reporting the highest levels of pain. Mean pain was not significantly related to SLA at Late Practice 2 (ρ = 0.207, *P* = 0.542) or Retention epochs (ρ = −0.377, *P* = 0.253).

### Experiment 2 (H3)

**Figure 6.**
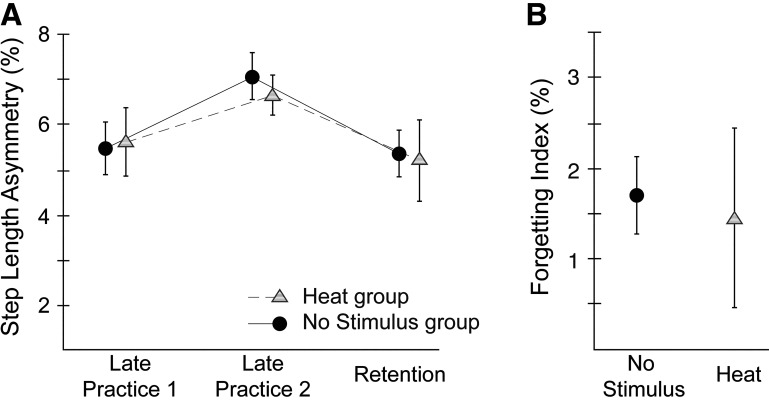
*Experiment 2*. Group average step length asymmetry values (SLAs) at Late Practice 1, Late Practice 2, and Retention epochs (*A*), and group average forgetting indices for the Heat Only (triangles) and No Stimulus (circles) groups (*B*). Error bars indicate ±1 SE.

An additional 12 individuals in the Heat Only group completed the locomotor learning task while experiencing a nonpainful heat stimulus during the learning on *day 1*. All participants in the Heat Only group rated their pain as 0 at all time points. Figure 6 shows group average SLAs for No Stimulus and Heat Only groups during the Late Practice and Retention epochs (Fig. 6*A*) and group average forgetting indices (Fig. 6*B*). The 2 × 3 (group × epoch) ANOVA indicated there was no significant interaction [*F*(2,42) = 0.12, *P* = 0.884, $\eta_{\text{p}}^{2}$ = 0.006] and no main effect of group [*F*(1,21) = 0.04, *P* = 0.839, $\eta_{\text{p}}^{2}$ = 0.002]. As expected, there was a significant main effect of epoch [*F*(2,42) = 5.0, *P* = 0.011, $\eta_{\text{p}}^{2}$ = 0.192]. There was also no difference in forgetting indices between the two groups [*t*(14.87) = 0.23, *P* = 0.819]. Thus, a heat only stimulus did not result in any changes to either the acquisition or retention component of locomotor learning.

## DISCUSSION

We compared the acquisition and retention of a novel, asymmetric stepping pattern learned during treadmill walking between a group of participants who experienced acute, tonic cutaneous pain and a group who experienced no stimulus. We found that, whereas the amount learned on *day 1* did not differ between the Pain and No Stimulus groups, the learned asymmetry remembered at the beginning of *day 2* was reduced for the Pain group compared with the No Stimulus group, indicating impaired 24-h retention in the Pain group. Furthermore, the degree of forgetting from *day 1* to *day 2* was also significantly more for the Pain group than the No Stimulus group. Finally, the amount of pain reported by participants in the Pain group during learning on *day 1* was positively correlated with the amount of forgetting experienced between *days 1* and *2* (pain intensity predicted ∼47% of the variance in forgetting). In *experiment 2*, we demonstrated that a control stimulus of heat without pain had no such effect on retention or forgetting. To our knowledge, this is the first study to show that tonic, cutaneous pain, but not other somatosensory stimuli, affects retention but not the initial learning of a new locomotor pattern and the first to show a correlation between pain levels and impaired motor memory consolidation.

### Pain and Acquisition of Motor Learning

As we expected, we did not see any differences in learning acquisition between the Pain and No Stimulus groups. A similar lack of effect of pain on motor learning acquisition has been reported by other studies that examined a walking task with either a topical (31, 45) or intramuscular (46) pain stimulus. The present results, together with these studies, suggest that acute pain may not hinder locomotor learning acquisition, generally. In the present study, we selected a cutaneous painful stimulus that could not be changed or avoided by selecting an alternative motor execution strategy. This appears to have been successful in avoiding any potential confounds due to altered motor performance and/or changes in baseline stepping patterns between groups. Therefore, we think this specific motor learning paradigm combined with our capsaicin and heat pain stimulus represents a rigorous model to isolate the effects of acute pain on motor learning acquisition.

### Pain and Retention of Motor Learning (H1)

Interestingly, on *day 2*, individuals in the Pain group did not immediately reproduce the walking asymmetry task as well as the No Stimulus group did, indicating reduced retention of the newly learned walking pattern. Because this was tested in a pain-free state, this finding cannot be due to any direct effects of pain on walking in *day 2*. Also, as discussed earlier, this was not reflective of differences in *day 1* acquisition or baseline walking in the painful state, as we observed no such effects. Instead, we hypothesize that the reduced retention and increased forgetting between sessions is due to a disruptive effect of pain on motor memory consolidation, possibly due to neuromodulatory effects of pain on M1. M1 is a key player in motor learning processes and, in particular, in consolidation of newly learned motor patterns. For example, practicing a new movement is accompanied by increases in cortical excitability of M1 (47, 48). In addition, increasing excitability in M1 via noninvasive brain stimulation improves motor learning consolidation (22), whereas decreasing excitability in M1 reduces retention (23, 25, 49). Acute pain, such as the experimental pain we induced with capsaicin and heat, has been shown to reduce corticomotor excitability in M1 (12, 19–21, 50, 51). Based on this literature, we posit that the acute pain stimulus used in our study interfered with consolidation of the practiced walking pattern, possibly by decreasing corticomotor excitability. Future studies should directly test this hypothesis.

An alternative explanation for the findings of *experiment 1* is that the painful stimulus causes some sort of distraction or divided attention effect that manifests during retention. Importantly, the effects of divided attention on motor learning have been studied extensively. Although distraction has been reported to disrupt initial learning acquisition in various ways (52–58), effects isolated to consolidation or retention have not been observed (59, 60). Nevertheless, the motivation behind *experiment 2* was to address this possibility, given that, to our knowledge, no other studies of pain and motor learning have tried to control for an effect of distraction or attention using a similar somatosensory but nonpainful stimulus. As anticipated (H3), we found no differences in locomotor learning acquisition, retention, or forgetting between the Heat Only and No Stimulus groups. This result suggests that the effects of pain on consolidation but not acquisition of motor learning are not generalizable to any sensation, but rather, specific to the sensation of pain. However, it should be noted that, because pain is a complex, multifaceted experience not limited to somatosensation (61), it may not be possible to fully disentangle pain from other cognitive and emotional processes such as attention, distraction, anxiety, and fear. That is, with respect to the current results, because they experienced pain, the Pain group may have experienced greater attentional demands than the Heat Only group. The possibility of pain-related cognitive and emotional factors playing a role in motor learning capacity should be explored in future work. Potential neural correlates that may support such a role would likely include the anterior and middle cingulate cortices (62–64), insula (62–64), and amygdala (65, 66).

Another explanation for our findings is that the increased forgetting is actually an effect of a change in context, rather than an effect of impaired consolidation (45). For the Pain group, the new walking pattern was acquired in the presence of pain, and then retention was tested in a pain-free context, whereas the No Stimulus group both practiced and was tested for retention in a pain-free context. One similar study did show an apparent effect of this kind of context change, from a painful to a nonpainful state, in reducing motor learning retention (45). We cannot definitively rule out this possibility, but we think there are several findings that suggest this is not a likely explanation for our results. First, several other studies that have examined learning acquisition in a painful context and retention in a pain-free context have not seen that this change in context impacts retention (30, 32, 34, 46, 67). Second, in the present study, we did not find that a change in context for the Heat Only group led to any deficiency in retention. Finally, the *catch* period during the *learning 1* block on *day 1* gives us some additional insight into this question. Recall that the *catch* period was inserted between *practice* *periods 1* and *2* to measure aftereffects present when participants briefly returned to normal walking without any visual feedback. Such an aftereffect is taken as an indicator of implicit central nervous system (CNS) storage (i.e., immediate retention) of the newly learned walking asymmetry (36, 37). When we compare the size of the *catch* period aftereffect between groups, we see that the No Stimulus group had an aftereffect of about 1.52% (±2.03 SD), which is nearly twice that of the Pain group’s aftereffect of 0.79% (±1.20 SD). This difference is not statistically significant here [*t*(12.4) = −0.956, *P* = 0.36], but this is likely only because acquisition was incomplete at this stage, making the aftereffects also incomplete. If that is the case, this is an indicator that the Pain group may have had less storage of the new walking pattern already evident during *day 1*. Importantly, the *catch* trial was performed in the painful state for the Pain group so there was no context change. Along the same lines, we can also look at how much was forgotten by each group after the *catch* period; i.e., between the end of *practice 1 period* and the beginning of *practice 2 period*. We can think of this as a within-session measure of forgetting, similar to our between-session forgetting index. Here, within-session forgetting for the Pain group was 1.3% ± 2.3 whereas there was no forgetting for the No Stimulus group (−0.19% ± 2.3). Although again not statistically significant [*t*(20) = 1.5, *P* = 0.158], we think this and the aftereffect observation suggest that the difference between groups in between-session forgetting was not due to an effect of a change in context.

Our results differ from a few other studies that did not see any effects of acute pain on motor learning retention. There are several differences between our study and these, any of which may have contributed to the difference in findings. These include the type of pain stimulus, the pain intensity, the task to be learned, and the control of motor performance confounds (30, 32, 34, 46). On the other hand, our results are in agreement with Bouffard et al. (31) who used a similar pain stimulus and also examined a walking task. They found that when participants walked with a plantarflexion force externally applied during the swing phase of gait, experiencing cutaneous pain did not affect how well they were able to adapt to counter the force and return to their normal ankle kinematics, but it did interfere with their performance of the task 24 h later. The present study extends these findings by addressing the effect of attention/distraction, specifically testing for equivalency between groups at baseline, and by using an explicit, strategic motor learning paradigm. Our paradigm closely resembles the type of learning used in physical rehabilitation where patients are given verbal instructions and performance-based feedback to train a motor pattern that is different from their baseline movement. Notably, there may be important differences in the effects of pain on various forms of learning, because of the different brain regions recruited for different forms of learning and retention. For example, we speculate that the cognitive and attentional demands of the current paradigm may be aspects that are particularly sensitive to effects of pain.

### Pain Intensity Is Associated with Forgetting of Locomotor Learning (H2)

We also showed, for the first time, a relationship between perceived pain intensity and the amount of between-session forgetting. Notably, this relationship was not present between pain and SLA retention, indicating that it is not the performance during retention testing that is predicted by pain levels, but specifically, the amount of learning that was lost between *days 1* and *2*. This is exactly what would be expected from an effect of pain on the motor memory consolidation process. Though this result will need to be replicated in a larger sample, it is also applicable to rehabilitation interventions because it suggests there may be a dose-response effect and that measures to reduce pain in clients prior to motor learning interventions may be helpful, even if pain cannot be fully ameliorated. Also of note, pain levels did not relate to locomotor learning acquisition, consistent with our finding of no effect of pain on acquisition.

### Conclusions

Our findings have two important conclusions. First, acute pain experienced during explicit, strategic locomotor learning reduces 24-h retention of a newly learned walking pattern. Second, the degree of retention loss is related to the perceived pain level during learning. There are potentially important implications of this work for physical rehabilitation interventions: pain felt during a motor learning-based intervention may lead to reduced retention of the new movement pattern during follow-up sessions, at least initially, even though it may not appear to interfere with the initial learning. Future work should investigate whether the relationship between pain level and retention of motor learning is a causal one and whether the effect can be overcome with repeated practice.

## DATA AVAILABILITY

Data will be made available upon reasonable request.

## GRANTS

This work was supported by the National Institutes of Health Grants R01-AG071585 (to S.M.M.), T32-HD007490 (to J.E.G.), S10-RR028114 (to S.M.M.), and F31-NS111806 (to M.A.F.), and by the Foundation for Physical Therapy Research (to J.E.G.).

## DISCLOSURES

No conflicts of interest, financial or otherwise, are declared by the authors.

## AUTHOR CONTRIBUTIONS

J.E.G. and S.M.M. conceived and designed research; J.E.G. and M.A.F. performed experiments; J.E.G. and S.M.M. analyzed data; J.E.G. and S.M.M. interpreted results of experiments; J.E.G. prepared figures; J.E.G. drafted manuscript; J.E.G. and S.M.M. edited and revised manuscript; J.E.G., M.A.F., and S.M.M. approved final version of manuscript.
